# The wound healing effect of polycaprolactone-chitosan scaffold coated with a gel containing Zataria multiflora Boiss. volatile oil nanoemulsions

**DOI:** 10.1186/s12906-024-04352-1

**Published:** 2024-01-25

**Authors:** Mahmoud Osanloo, Fariba Noori, Negar Varaa, Alireza Tavassoli, Aida Goodarzi, Maryam Talebi Moghaddam, Lida Ebrahimi, Zahra Abpeikar, Ahmad Reza Farmani, Mohsen Safaei, Narges Fereydouni, Arash Goodarzi

**Affiliations:** 1 Department of Medical Nanotechnology, School of Advanced Technologies in Medicine, Fasa University of Medical Sciences, Fasa, Iran; 2 Department of Tissue Engineering, School of Advanced Technologies in Medicine, Fasa University of Medical Sciences, Fasa, Iran; 3 Department of Anatomy, School of Medicine, Fasa University of Medical Sciences, Fasa, Iran; 4 Department of Pathology, School of Medicine, Fasa University of Medical Sciences, Fasa, Iran; 5 Noncommunicable Diseases Research Center, Fasa University of Medical Sciences, Fasa, Iran; 6 Student Research Committee, Fasa University of Medical Sciences, Fasa, Iran

**Keywords:** Wound healing, Zataria multiflora, Volatile Oil nanoemulsion, Hydroxypropyl methylcellulose (HPMC), Polycaprolactone (PCL), Chitosan

## Abstract

**Aims:**

Thymus plant is a very useful herbal medicine with various properties such as anti-inflammatory and antibacterial. Therefore, the properties of this plant have made this drug a suitable candidate for wound healing. In this study, hydroxypropyl methylcellulose (HPMC) gel containing Zataria multiflora volatile oil nanoemulsion (neZM) along with polycaprolactone/chitosan (PCL-CS) nanofibrous scaffold was used, and the effect of three experimental groups on the wound healing process was evaluated. The first group, HPMC gel containing neZM, the second group, PCL-CS nanofibers, and the third group, HPMC gel containing neZM and bandaged with PCL-CS nanofibers (PCL-CS/neZM). Wounds bandaged with common sterile gas were considered as control.

**Methods:**

The nanoemulsion was synthesized by a spontaneous method and loaded into a hydroxypropyl methylcellulose (HPMC) gel. The DLS test investigated the size of these nanoemulsions. A PCL-CS nanofibrous scaffold was also synthesized by electrospinning method then SEM and contact angle tests investigated morphology and hydrophilicity/hydrophobicity of its surface. The animal study was performed on full-thickness skin wounds in rats, and the process of tissue regeneration in the experimental and control groups was evaluated by H&E and Masson's trichrome staining.

**Results:**

The results showed that the nanoemulsion has a size of 225±9 nm and has an acceptable dispersion. The PCL-CS nanofibers synthesized by the electrospinning method also show non-beaded smooth fibers and due to the presence of chitosan with hydrophilic properties, have higher surface hydrophobicity than PCL fibers. The wound healing results show that the PCL-CS/neZM group significantly reduced the wound size compared to the other groups on the 7th, 14th, and 21st days. The histological results also show that the PCL-CS/neZM group could significantly reduce the parameters of edema, inflammation, and vascularity and increase the parameters of fibrosis, re-epithelialization, and collagen deposition compared to other groups on day 21.

**Conclusion:**

The results of this study show that the PCL-CS/neZM treatment can effectively improve wound healing.

## Introduction

Acute and chronic wounds are one of the major and fundamental problems in society's healthcare system, causing high financial and social burdens [1]. Autologous and allograft transplantation can be considered the gold-standard treatment options currently available for wound healing. However, due to the lack of access to healthy donors and immune response, they face many limitations [2–4]. Nevertheless, the emergence of tissue engineering, which is based on the use of scaffolds, stem cells, and growth factors, has led to the creation of a new generation of wound dressings that have the potential to significantly improve the healing process [5–7]. Therefore, given the progress in the synthesis of various biomaterials particularly polymeric biomaterials in recent years, the construction of engineered scaffolds for wound healing is considered an alternative to grafting methods [8–10]. Amongst, scaffolds synthesized by phase separation, self-assembly, and electrospinning techniques are the most effective methods to mimic the biological and physical properties of healthy skin [11, 12].

Electrospinning is a simple and highly effective technique for producing micro and nanofibers from natural and synthetic polymers. Compared with other 3D nanostructures, electrospun nanofibers can simulate the extracellular matrix of the natural skin and create suitable physical and mechanical conditions for cell attachment, proliferation, and differentiation. The important factors of this high biocompatibility are associated with the high specific surface area and high porosity that prevent fluid accumulation and facilitate oxygen permeability [13–16]. However, none of the skin substitutes has been able to restore the characteristics of healthy skin completely. Therefore, selecting a suitable combination of materials to improve biological and mechanical properties seems necessary [17–20].Since the use of a single polymer in a scaffold may not meet all expected requirements for biocompatibility and mechanical properties, researchers are interested in using systems based on the combination of two or more polymers [21–24].

Chitosan (CS) is an abundant polymer with advantages such as biocompatibility, biodegradability, antibacterial and blood coagulation properties, so it seems ideal for wound healing applications. However, due to the polycationic nature of the chitosan solution and its rigid chemical structure, its electrospinnability is limited [25]. Therefore, it combines with other polymers such as polyvinyl alcohol (PVA), polyethylene oxide (PEO), or polycaprolactone (PCL) to overcome this limitation [26–28]. Polycaprolactone is a biocompatible and biodegradable polymer that can produce suitable electrospun scaffolds and exhibits good mechanical strength in aqueous environments [29, 30]. As a result, the combination of chitosan with PCL improves its electrospinning ability to obtain nanofibrous scaffolds. Studies reveal that the PCL-CS combined electrospun scaffolds exhibit desirable properties such as mechanical strength, controlled degradability, water retention, and admissible wound healing [31, 32].

On the other hand, applications of sustained drug delivery systems in regenerative medicine, especially in wound healing, have increased drastically [33–36]. Meanwhile, the use of herbal medicinal extracts in wound healing has attracted the attention of many researchers due to its advantages, such as few side effects, ease of extraction, and reasonable price [37, 38]. Amongst, Zataria Multiflora Boiss (ZM), called in Persian "Shirazi thyme", has anti-inflammatory, antioxidant, and immunomodulatory effects, making it a suitable option for use in wound healing scaffolds [39–41]. This is a plant species belonging to the Lamiaceae family and is native to Southeast Asia. However, it mainly grows in the central and southern regions of Iran, Pakistan, and Afghanistan [42]. It is beneficial in treating cramps-associated pains [43], dysmenorrhea [44], indigestion [45], nausea, diarrhea [46], and infectious diseases [47, 48]. Also, pharmaceutical researches demonstrated that this plant has antifungal and antimicrobial properties [45, 49, 50]. Particularly, the essential oil of this plant has long been used for bacterial infections [51–53]. Previous studies have demonstrated that the antibacterial activity of this plant can be attributed to phenolic compounds such as thymol and carvacrol. These compounds exhibit antibacterial properties by destroying cell walls and membranes [50, 52].

Despite the particular medicinal properties of this plant in accelerating wound healing, few studies have investigated the properties of wound healing. For instance, Farahpour et al. showed that Zataria Multiflora essential oil with anti-inflammatory and antibacterial properties improves wound healing compared to the control group [54]. Also, Farahani et al. also reported significant wound healing properties of Zataria Multiflora essential oil loaded in the cellulose acetate/gelatin nanofiber scaffold [55].

Additionally, regarding the great potential of nanoemulsion formulations in drug delivery, their application in preparing novel scaffolds could be promising [56, 57]. Meanwhile, considering the desirable properties of hydroxypropyl methylcellulose (HPMC), such as safety, hydrophilic nature of gel matrix, and excellent skin biocompatibility, it can be considered as a good candidate for preparing nanoemulsion formulation [58–61].

Subsequently, it seems that using plant extracts in nanoemulsion formulation along with nanofiber dressings in wound healing could be a promising novel approach for improving wound healing. However, few studies have investigated the use of this plant and plant extracts in nanoemulsion formulation along with nanofiber dressings in wound healing. For example, in a study, a nanoemulsion of Zataria Multiflora (neZM) was loaded in cellulose acetate/gelatin nanofibers, and promising results in improving wound healing were observed [55]. Hence, In this study, neZM was loaded in an HPMC gel substrate. This gel matrix is then coated on the PCL-CS scaffold to provide a suitable scaffold for wound healing.

## Material & methods

### Materials

The materials used in this project were purchased as the following: polycaprolactone (PCL) (Sigma–Aldrich, Germany), chitosan (Easter Holding Group, China, MW: 100 KDa, deacetylation degree: 93%), Zataria Multiflora volatile oil (ZMVO) (Zardband Pharmaceuticals Co, Iran). Also, Tween 20, Tween 80, and span 80, Hydroxypropyl Methylcellulose (HPMC), Hexafluoro-2-propanol (HFIP), Hematoxylin, and Eosin stain (H&E) were bought from Merck, Germany. Glacial acetic acid and ethanol (>99.7 %, Dr. Mojallali, Iran), and Masson trichrome staining kit (Asiapajohesh, Iran). Deionized water (DW) has been applied in all involved experiments.

### Synthesis and characterization of nanogel containing Zataria multiflora nanoemulsions (neZM)

The Oil in Water (O/W) nanoemulsion of ZM volatile oil (neZM) was synthesized by the spontaneous method according to the previous report [62]. Briefly, ZMVO (1% v/v), surfactants of tween 20 (2% v/v), tween 80 (2% v/v), and span 80 (1% v/v) were mixed completely to form a homogenous solution using a stirrer (1500 rpm) in room temperature (RT) for 10 mins. In the second step, the deionized water was added dropwise and mixed (1500 rpm, 30 min).

The droplet size and droplet size dispersity of the prepared neZM were investigated using a dynamic light scattering (DLS) type apparatus (K-One NANO- Ltd. Korea). D50 (median diameter of particles at 50 cumulative percent) was considered as droplet size and droplet size dispersity was calculated using the below equation:

$$Droplet\ size\ dispersity=\sqrt{d75}\div d25$$

### Preparation and characterization of electrospun PCL-CS NFs

PCL-CS nanofibers were prepared by electrospinning methods as described in a previous report, with some minor modifications [63]. Briefly, PCL (14% w/v), and CS (1% w/v) solutions were prepared using hexafluoroisopropanol (HFIP); then, the solutions were mixed with a ratio of 1:3 (v/v), respectively. The prepared polymeric solution was transferred into a 10-mL syringe (15 mm internal diameter) and attached with a blunted metal needle (22 G). The syringe was situated in a syringe pump in the electrospinning machine (Fanavaran Nano-Meghyas, FNM Co. Ltd, Iran). Instrumental factors were set as follows: 0.8 mL/h injection rate, 15 kV applied DC voltage, and 100 mm distance between the needle and rotating cylindrical collector (100 rpm). A thin layer of aluminum foil was wrapped on the collector to facilitate the separation of the fabricated nanofibers.

The diameter and morphology properties of the nanofibers were examined using scanning electron microscopy (SEM; Vega 3, TESCAN Co, Czech Republic). The diameter size distribution of NFs was measured using Image-J software (https://imagej.net/Fiji) along with the sample size of 100 NF. The contact angle of the water (θ) with the surface of the nanofibers was measured using a contact angle measurement system (CA-500A, Sharif Solar Co. Iran). Attenuated Total Reflectance-Fourier Transform Infrared spectroscopy (ATR-FTIR) spectra for powders of CS and PCL and electrospun NFs (PCL-CS) were recorded on a Tensor II instrument (Bruker Co. USA) in the range of 500-3500 cm-1.

### In vivo study

Sixty male Wistar rats, approximately two months old and weighing 200-250 g, were received from the Fasa Medical School animal house and used as a wound healing model. The rats were kept in polystyrene cages according to the conservation rules and respect for animal rights, with a light cycle of 12 hours, standard temperature conditions (25±2 °C), humidity, and free access to food and water. All procedures and experiments involving animals were approved by the Bioethics Committee of Fasa University of Medical Sciences (Ethics code: IR.FUMS.AEC.1401.012) and performed in accordance with the guidelines for the care and use of laboratory animals in Iran. All methods are reported according to the ARRIVE guidelines.

**Fig. 1:**
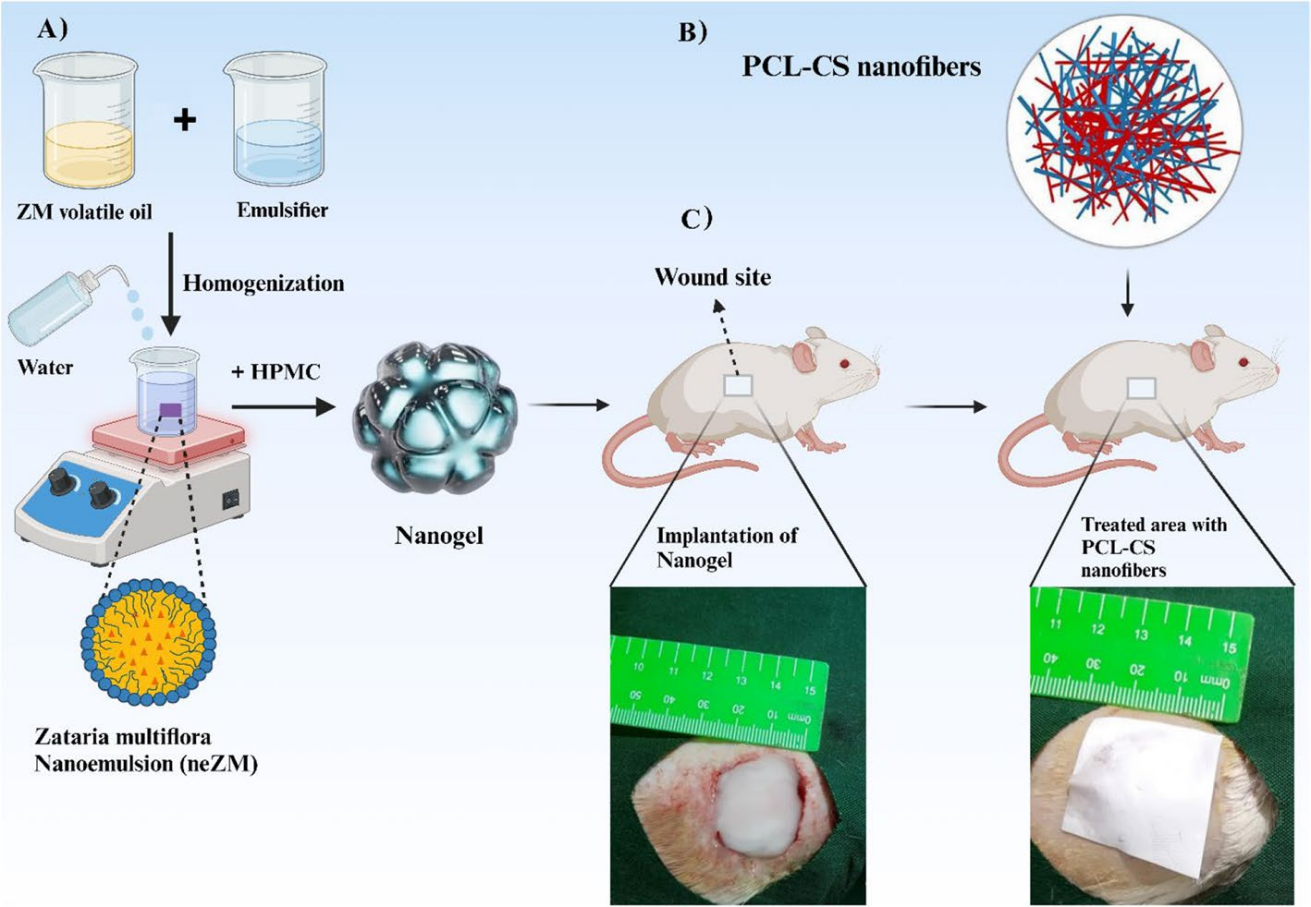
The processes of study. **A** Synthesis of hydroxypropyl methylcellulose (HPMC) gel containing Zataria multiflora volatile oil nanoemulsion (neZM); (**B**) fabrication of fibrous scaffolds by electrospinning technique; (**C**) implantation of nano-gel in the lesion site and bandage of the treated site by PCL-CS nanofibers. Created in BioRender.com

**Fig. 2:**
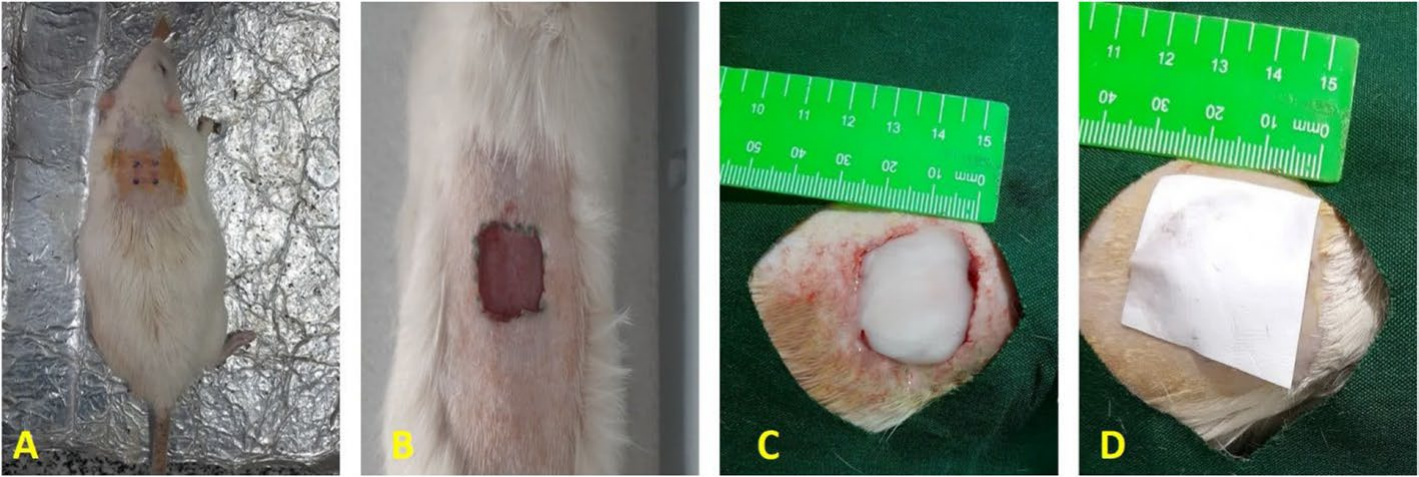
**A** Surgical procedure on the skin of the rat, shaving the dorsal area of the rat. **B** generating a square-shaped wound with full-thickness excision measuring 20 mm × 20 mm on the rat's dorsal skin. **C** Implantation of hydroxypropyl methylcellulose gel containing Zataria multiflora volatile oil nanoemulsion into the wound site (**D**), covering the treated area with polycaprolactone/chitosan nanofibers

Before the experiments, the cages and chambers were disinfected with Dettol and then exposed to UV radiation for 24 hours to create a pathogen-free environment. The rats were anesthetized with an intraperitoneal injection of ketamine-xylazine (70:30). Then, the animal's back hair was shaved and thoroughly cleaned at the wound site. The expected wound area was disinfected with a 10% Povidone Iodine solution. Then, using a stencil ruler, a 4 cm^2^ wound was created by a full-thickness surgical razor. Finally, the treatments were applied directly to the newly created wound (Figs. 1 and 2).

### Study groups

Rats were randomly divided into four groups. In each of the groups, there were five rats for the study on days 3, 7, and 14. The study groups were studied as follows.

A  Control group (wound washing with normal saline and bandage with common sterile gas)
B The rats treated with HPMC gels containing neZM (neZM group)
C The rats treated with nanofibers (PCL-CS group)
D The rats treated with nanofibers coated with HPMC gels containing neZM (PCL-CS/neZM group)

The same amount of 2.5 × 2.5 cm of PCL-CS coated with 2 ml of neZM was used for the respective groups. This value was selected for the nanofibers based on the size of the wound and for the neZM on how much it can cover the entire wound. Then, they were kept in separate cages and in a particular room until the end of the study.

### Macroscopic assessment of the wounds

The wound healing rate was assessed by measuring the length and width of the wound with a common ruler on days 7, 14, and 21. The wound closure area was performed in different groups with five repetitions, and the results were reported as mean ± standard deviation (SD).

### Histology study

After sacrificing the animals by CO2 euthanasia on day 21, the wound and approximately 5 mm surrounding it were excised with scissors and forceps to maximum thickness. The wound tissues were then fixed in 10% formalin solution, dehydrated in increasing concentrations of alcohol, embedded in paraffin, sectioned, and mounted on glass slides for Hematoxylin-Eosin and Masson trichrome examination and examination—finally, more in-depth histology. Qualitative assessment was performed according to our previous study [64].

### Statistical analysis

Statistical analyses were performed using GraphPad Prism 6.0 software. The normality of the data was measured by the Kolmogorov-Smirnov test. The qualitative data (Edema, inflammation, vascularity, fibrosis tissue, re-epithelialization, contracture, and Masson's trichrome staining scores) were converted to equivalent percentages. Therefore, one-way ANOVA and Tukey post hoc test were performed to compare the mean ± standard deviation (SD) for wound size and histopathological scores. The significance level in all analyses was considered less than 0.05 (p <0.05).

## Result

### Characterization of neZM and PCL-CS nanofibers

#### DLS of neZM

**Fig. 3:**
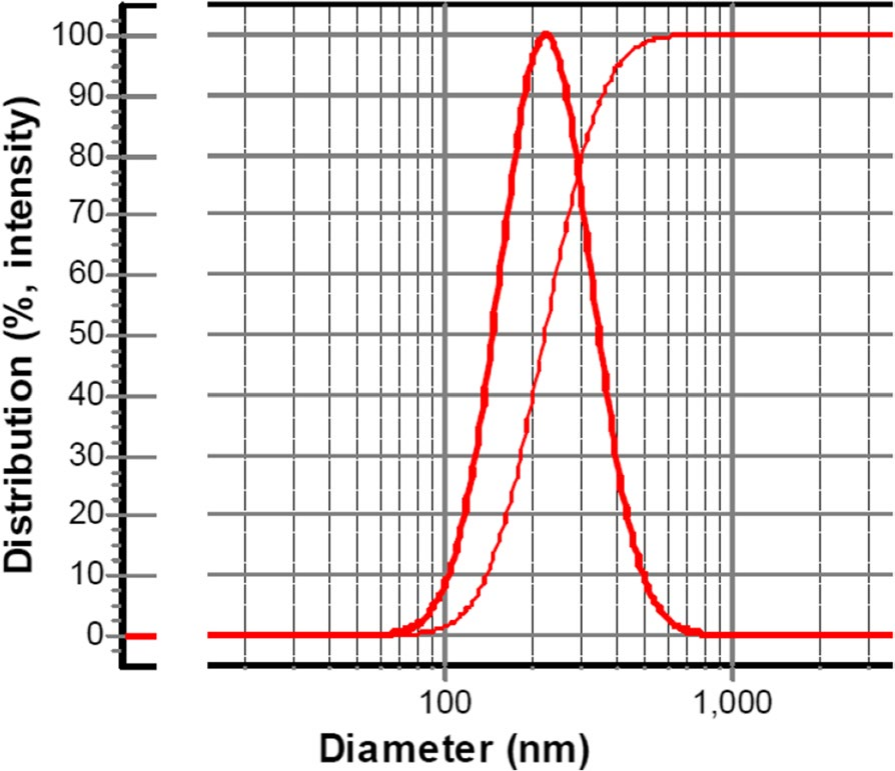
The droplet size of neZM was synthesized by a spontaneous method; the average droplet size was about 225 ± 9 nm and their PDI was 0.97

Nanoemulsion is a category of emulsions with very small and uniform droplet sizes of about 20 to 500 nm. Nanoemulsions have high kinetic stability, which makes them stable against precipitation and cream formation. The droplet size of a nanoemulsion is an important factor in evaluating the nanoemulsion. The droplet size of nanoemulsion is affected by the loaded drug, oil, and surfactant concentration [65–67]. This study used adjusted concentrations of ZMVO, Tween 20, Tween 80 and Span 80 surfactants to synthesize neZM.Figure 3 shows the droplet size of the synthesized nanoemulsion; its average size was calculated to be 225 ± 9 nm, and the PDI was within the acceptable range, i.e., 0.97. Therefore, it seems that the nanoemulsion synthesized in this study has uniform droplet size and its droplets are nanosized.

#### PCL-CS nanofibers

**Fig. 4:**
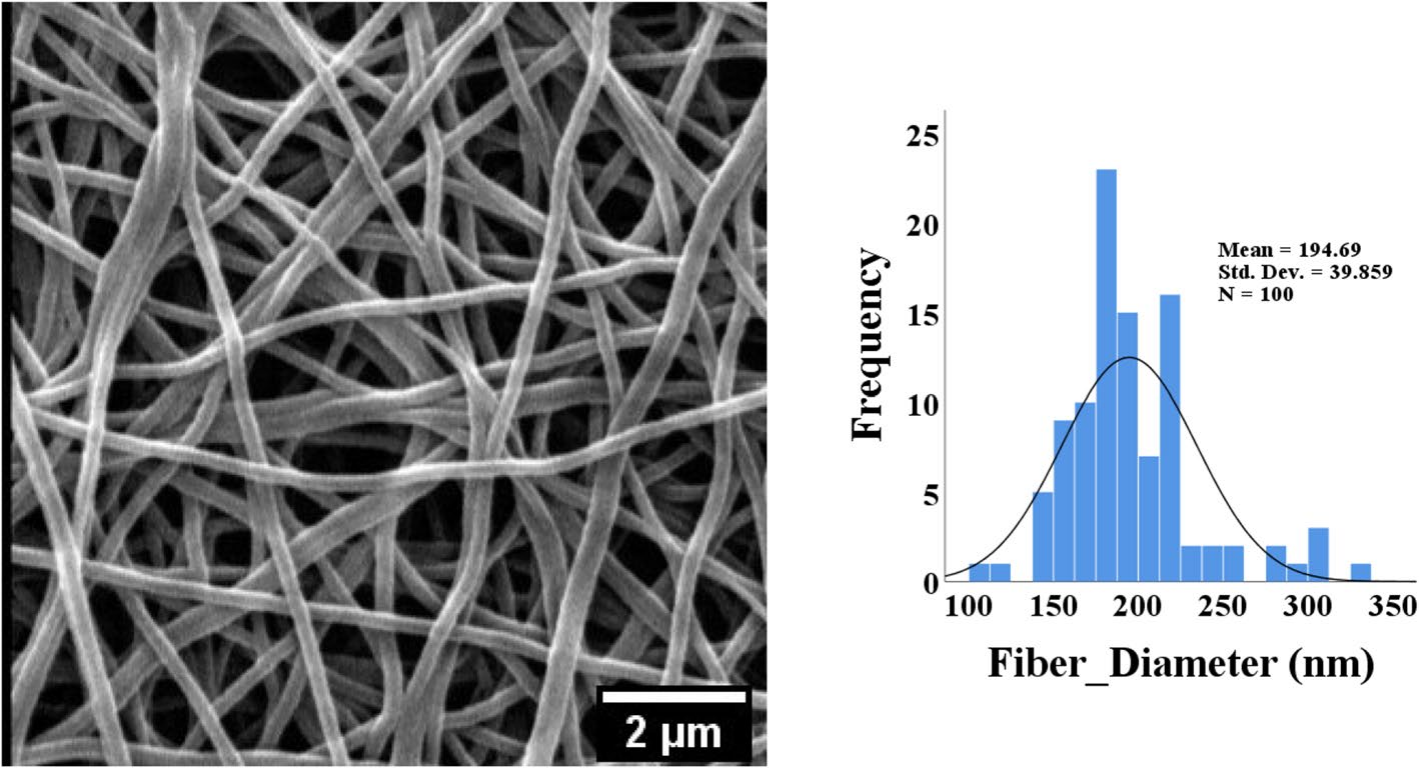
Morphology and size distribution of PCL-CS nanofibers, nanofibers have uniform size, and smooth morphology with a mean size of 194.7 ± 39.9 nm

One of the important characteristics of electrospun nanofibers is morphology because these nanometer-sized fibers mimic the structure of the natural extracellular matrix. Therefore, cells can completely adapt to the environment created by the scaffold and induce better growth, proliferation, and migration [68–70]. Figure 4 shows the SEM image of the nanofiber morphology. The figure shows that the synthesized nanofibers are grainless, smooth, and uniform, with an average size of 194.7 ± 39.9 nm.

**Fig. 5:**
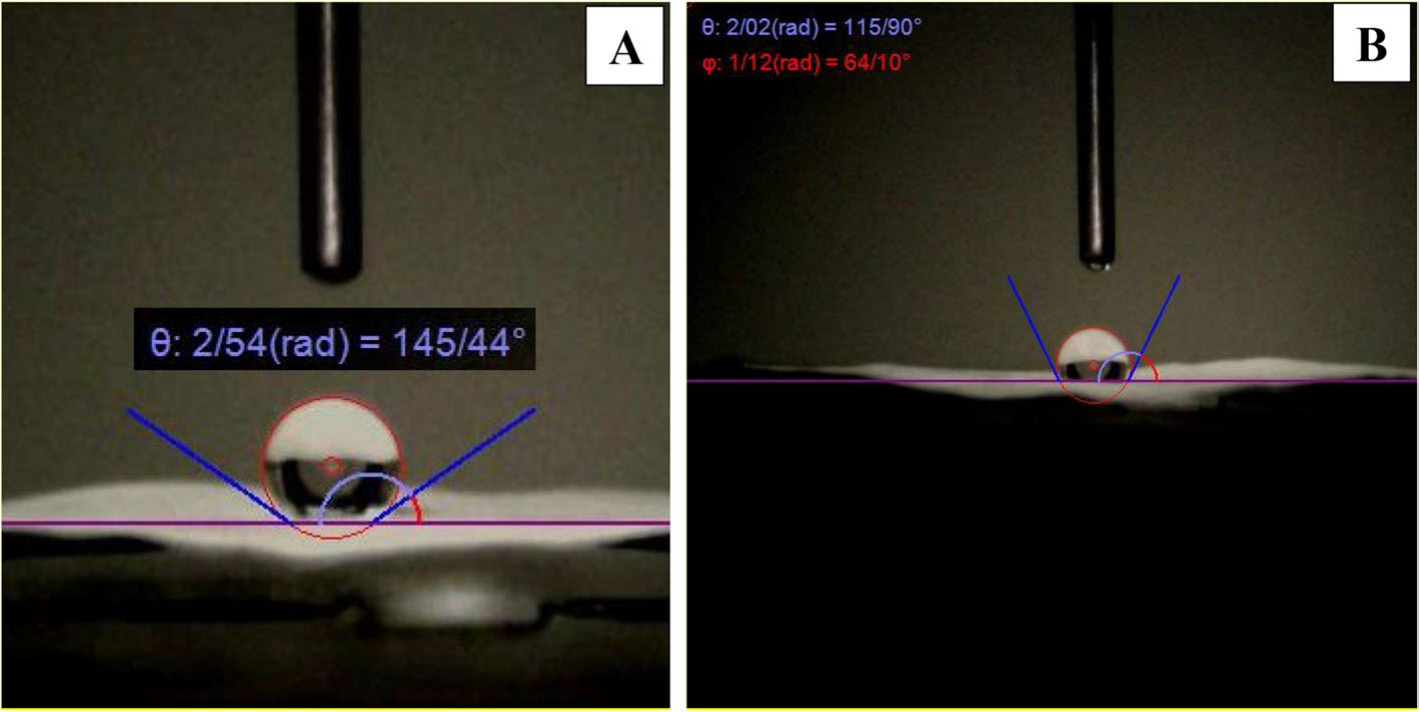
Surface hydrophobicity of nanofiber scaffolds, (**A**) PCL, and (**B**) PCL-CS

Also, another important factor of nanofiber scaffolds in tissue engineering is hydrophilicity, while nanofibers with acceptable hydrophilicity contribute to better cell adhesion and proliferation [71]. Figure 5A shows that the PCL nanofibers lead to a contact angle of 145.44°, which is due to the hydrophobic nature of this polymer. However, the addition of chitosan as a hydrophilic polymer due to OH groups has increased the hydrophilicity of nanofibers and decreased the contact angle of PCL-CS nanofibers to 115.9° (Fig. 5B).

#### FTIR

**Fig. 6:**
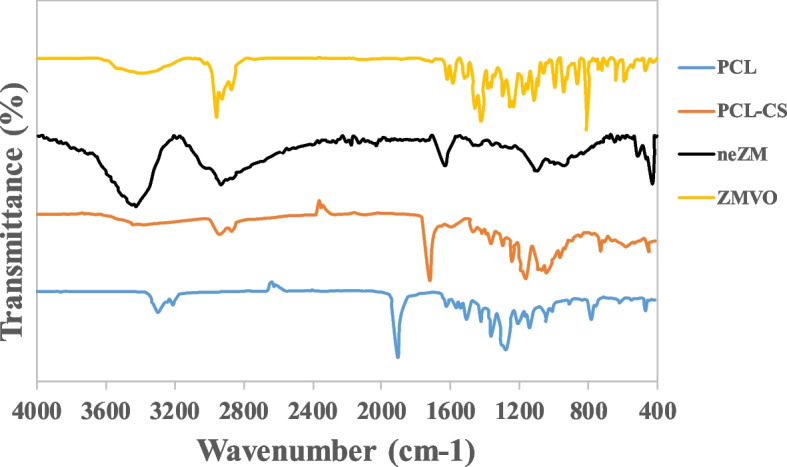
FTIR spectra of ZMVO, neZM, PCL, and PCL-CS nanofibers

The FTIR spectra of ZMVO, neZM, and PCL-CS nanofiber mat are shown in Fig. 6. The FTIR spectra of the ZMVO show a peak at 3386 cm^−1^, corresponding to hydroxyl groups. Also, the observed peaks at 2956 cm^−1^ and 2869 cm^−1^ can be related to the vibration of the bond C=H in the aromatic ring. Also, the peaks at wavelengths of 1400 cm^−1^ to1600 cm^−1^ may related to the C═C‒C ring vibration. Eventually, there is a distinctive peak at 809 cm^−1^, which is an indicator peak of thymol [72–74].

The FTIR spectra of the neZM shows HPMC-related peaks at 3421 cm^-1^ (–OH stretching), 2924 cm^-1^ (-CH stretching), 1629 cm^-1^ (carbonyl group), 1091 cm^-1^ (-C-O- stretching vibrations), and 935 cm^-1^ (pyranose ring) [75]. However, in the FTIR spectrum of neZM, the indicator peak of thymol was not visible, which demonstrated complete encapsulating of neZM nanoemulsions *via* HPMC nanogels.

The polycaprolactone spectrum revealed peaks related to -C-H at about 2943 cm^-1^ and 2865 cm^-1^, and a very sharp signal band may correspond to the carbonyl group at 1721 cm^-1^ [76, 77]. With adding chitosan to PCL, several new peaks have appeared in PCL-CS, which confirms the addition of CS to PCL. The broad index band around 3367 cm^-1^ is related to the stretching vibrations of -NH2 groups and the high degree of deacetylation of CS [78]. Peak 1590 is related to carbonyl stretching of amide bonds and N-H bending of CS amino groups and the fingerprint band of chitosan corresponding to C-N has appeared at 1041 cm^-1^ [79, 80]. The lack of observation of a new peak in nanofibers can indicate the existence of a simple combination between them so that no chemical bonds have occurred.

### Wound size

**Fig. 7:**
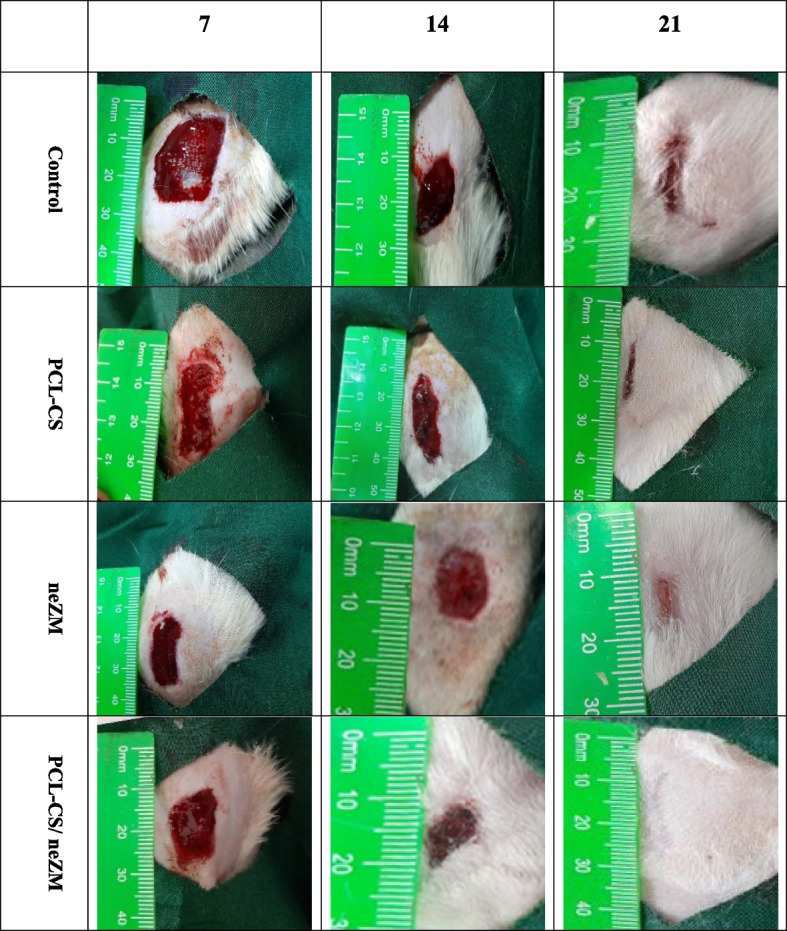
Macroscopic photograph of wound size in all groups on the 7th, 14th, and 21st days. The PCL-CS/ neZM treatment group clearly has lower wound size than all groups in all time intervals

**Fig. 8:**
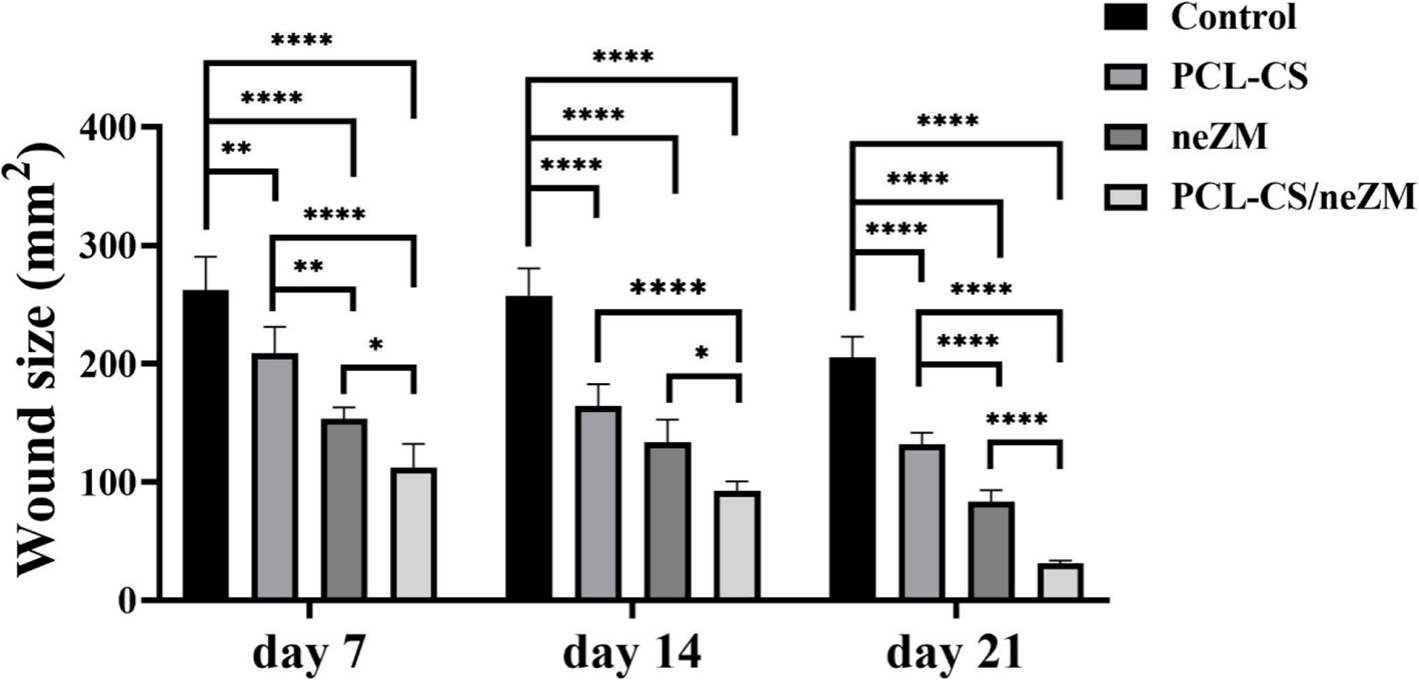
Statistical analysis of wound sizes on days 7, 14, and 21. *: *p*<0.05, **: *p*<0.01, and ***: *p*<0.001 shows the difference of significance level between the groups. Control: Control group, PCL-CS: PCL-CS nanofiber group, neZM: HPMC gels containing neZM group, PCL-CS/neZM: PCL-CS nanofibers coated with HPMC gels containing neZM group

Figure 7 shows the photograph of the wounds in all of the animal groups of this study on the 7th, 14th, and 21st days, and Fig. 8 statistically evaluates the measurements taken in millimeters. The statistical results demonstrate that the wound size in the PCL-CS/neZM group significantly decreased compared to the other three groups in all time intervals. Furthermore, on days 7 and 21, wound size decreased significantly more in the neZM group than in the control group and the PCL-CS group. In addition, the wound size in the PCL-CS group was only lower compared to the control group on days 7, 14, and 21 (Fig. 7).

### Histological analysis

**Fig. 9:**
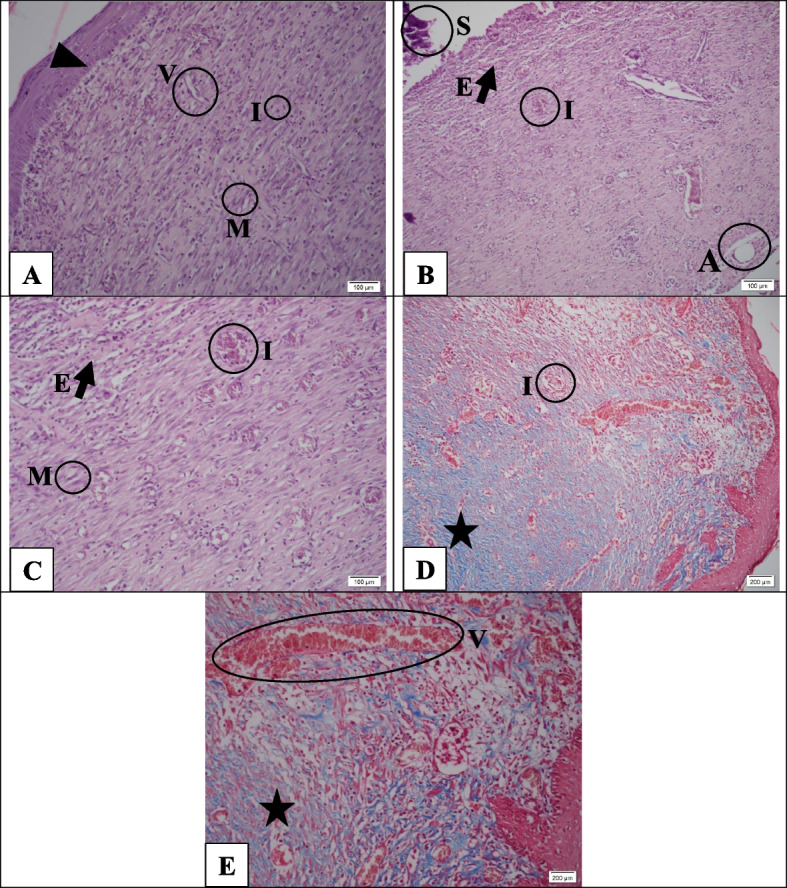
Hematoxylin-Eosin staining (**A**-**C**) and Masson's trichrome (**D**-**E**) in the control group. Although the epidermis is almost formed, inflammatory granulation tissue and scar can be observed. The guide of symbols and letters in the figure is as follows: Star sign: granulation tissue, Cross sign: fibrotic scar tissue, Head arrow: re-epithelialization, Arrow with letter E: edema, Letter I: inflammation, Letter A: micro-abscess, Letter V: vascularization, Letter M: myofibroblasts, Letter S: scab

Figure 9 presents tissue sections with H&E and Masson trichrome staining in the control group on day 21. Figure 9A shows that the epidermis is almost formed, and the wound is not visible. Also, the granulation tissue is not loose, and there is no micro**-**abscess, but moderate to high inflammatory cell infiltration is observed, which indicates the early stages and incomplete healing. Moreover, the *head arrow* represents re-epithelialization, and *Letter I* shows inflammatory cells (lymphocytes), in chronic inflammation in granulation tissue. Additionally, the *Letter V* also represents the new vessels in the granulation tissue. This tissue consists of new blood vessels and inflammatory cells. *Letter M* presents the myofibroblast cells in the superficial layers, indicating that the granulation tissue is in the midst stages of its development.

In Fig. 9B, the *Letter S* shows the scab, and the *Letter A* represents the abscess in the lowest layer of the wound. The *arrow with the letter E* reveals slight edema and inflammation. Also, Letter I demonstrates hyperemia congestion and chronic inflammatory cells in the middle of the tissue. Moreover, the same trend can be observed in Fig. 9C. In Masson trichrome sections, the black star indicates a middle phase of granulation tissue and scar formation (Fig. 9D and E).

**Fig. 10:**
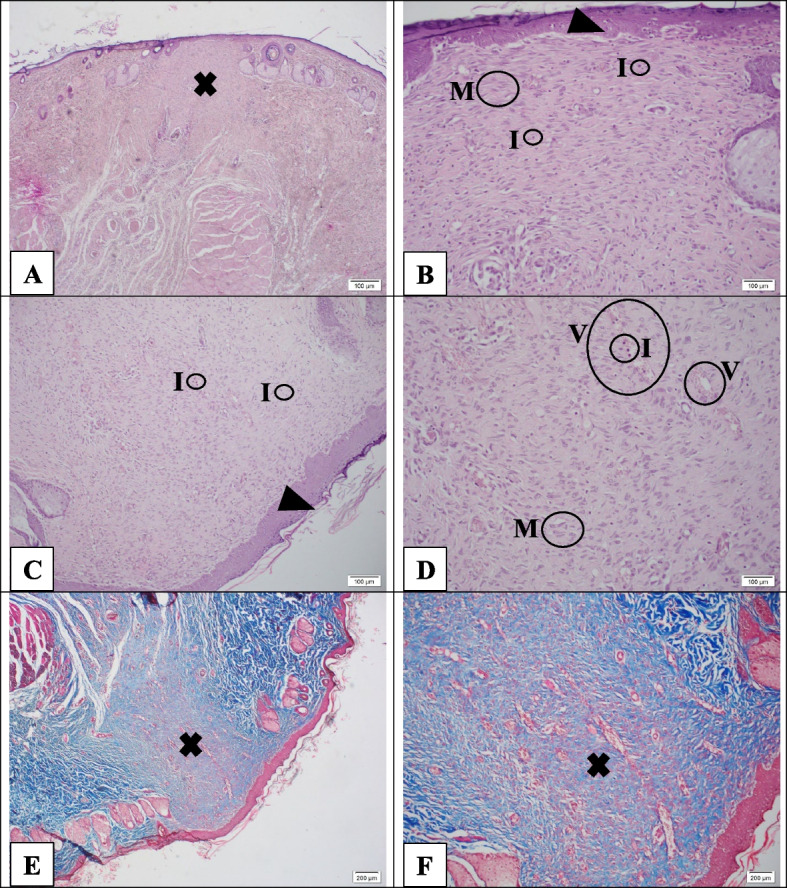
Hematoxylin-Eosin staining (**A**-**D**) and Masson's trichrome (**E**-**F**) in the neZM group. Although scar formation initiation was observed, its contracture has not yet been well done. The guide of symbols and letters in the figure is as follows: Star sign: granulation tissue, Cross sign: fibrotic scar tissue, Head arrow: re-epithelialization, Arrow with letter E: edema, Letter I: inflammation, Letter A: micro-abscess, Letter V: vascularization, Letter M: myofibroblasts, Letter S: scab

Figure 10 shows tissue sections with H&E and Masson trichrome staining in the neZM group on day 21. In Fig. 10A, *the cross sign* reveals pale pink tissue and a lack of vascularity and inflammation, indicating the onset of scar formation. Also, in Fig. 10B, the wound also begins to contract, and a thick epithelial layer has formed, but it has not reached the thickness of the epidermis of healthy skin (*Head arrow*). Moreover, *Letter I* represents a small number of monocytes and lymphocytes as inflammatory cells, and *Letter M* indicates abundant myofibroblast cells. Furthermore, in Fig. 10D, *Letter V* shows a few remaining blood vessels, indicating regeneration and scar formation. In Masson trichrome sections, the *cross sign* indicates scar tissue (Fig. 10E and F). In this group, the scar tissue contains an average number of blood vessels, and scar accumulation or contracture has not been formed well.

**Fig. 11:**
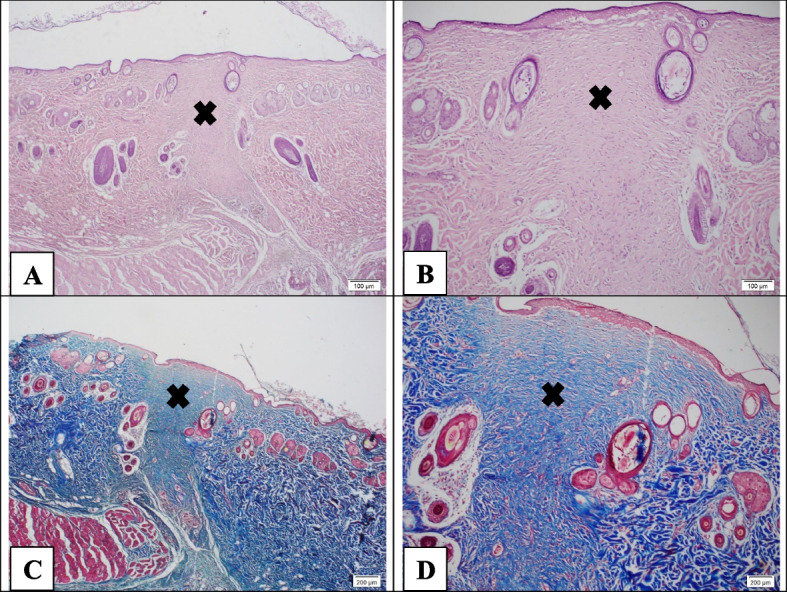
Hematoxylin-Eosin staining (**A**-**B**) and Masson's trichrome (**C**-**D**) in the PCL-CS group. The scar formation is in the final phase, but the skin appendages such as hair follicles and sebaceous are not appeared. Moreover, higher wound contracture was observed compared with the neZM group. The guide of symbols and letters in the figure is as follows: Star sign: granulation tissue, Cross sign: fibrotic scar tissue, Head arrow: re-epithelialization, Arrow with letter E: edema, Letter I: inflammation, Letter A: micro-abscess, Letter V: vascularization, Letter M: myofibroblasts, Letter S: scab

Figure 11 demonstrates H&E and Masson trichrome-stained tissue sections in the PCL-CS group on day 21. In this group, the scar is in the final stages or late phase. Blood vessels and inflammatory cells disappeared, as well as most of the myofibroblast cells transformed into spindle-shaped fibroblasts with irregular orientation. However, the skin appendages, such as hair follicles and sebaceous glands, are not seen in this part. The *cross sign* represents the scars in Fig 11A and B.

Additionally, in Masson trichrome sections, the *cross sign* indicates scar tissue (Fig. 11C and D). Wound contracture was better formed than the neZM group. The number of vessels has decreased, but the skin appendages are still not clear. The amount of connective tissue has increased, and abundant collagen has been deposited.

**Fig. 12:**
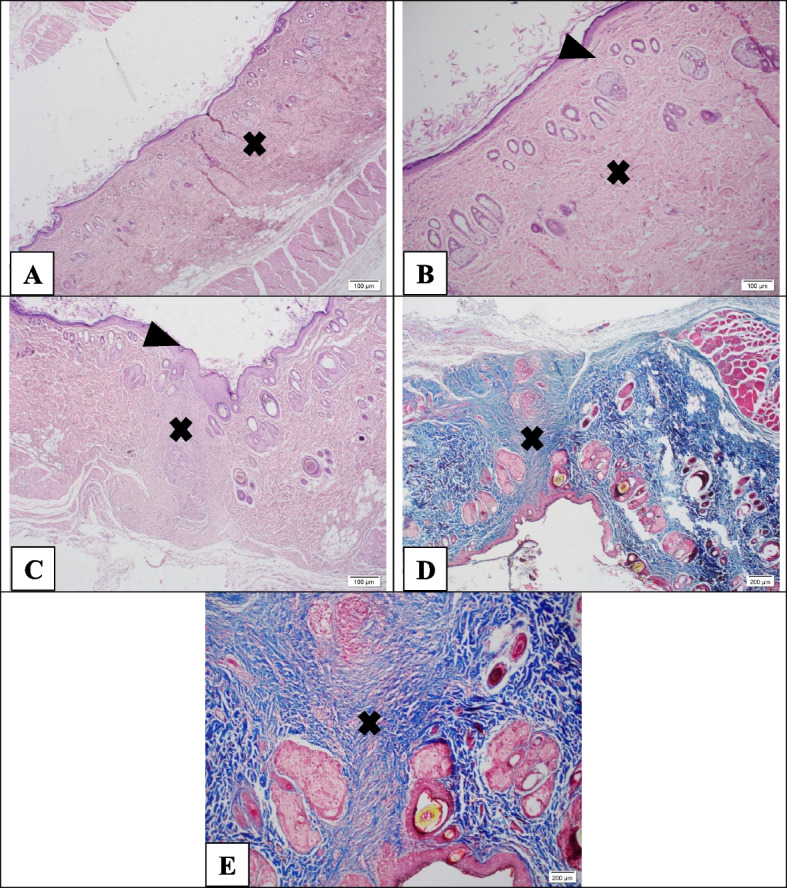
Hematoxylin-Eosin staining (**A**-**C**) and Masson's trichrome (**D**-**E**) in the PCL-CS/neZM group. Wound closure almost thoroughly happened, as well as skin appendages clearly appeared. The guide of symbols and letters in the figure is as follows: Star sign: granulation tissue, Cross sign: fibrotic scar tissue, Head arrow: re-epithelialization, Arrow with letter E: edema, Letter I: inflammation, Letter** A:** micro-abscess, Letter V: vascularization, Letter M: myofibroblasts, Letter S: scab

Figure 12 represents images of tissue sections with H&E and Masson trichrome staining related to the PCL-CS/neZM group on day 21. In Fig. 12A-C, the *head arrow* represents the epidermis of the wound site, which has completely regained the thickness of the previous epidermis. *The cross sign* reveals that the wound site has become very small due to the contracture caused by the scar, and the edges of the wound are completely close together. In the dermis of the scar tissue, a significant amount of matrix and a number of regular**-**laid myofibroblasts can be seen.

Moreover, in Masson trichrome sections, the *cross sign* indicates scar tissue (Fig. 12D and E). The scar tissue is completely contracted, so it is very difficult to identify the location of the wound in low magnification. The thickness of the epidermis has almost reached the thickness of the natural skin, and skin appendages can be seen on both edges of the wound.

**Fig. 13:**
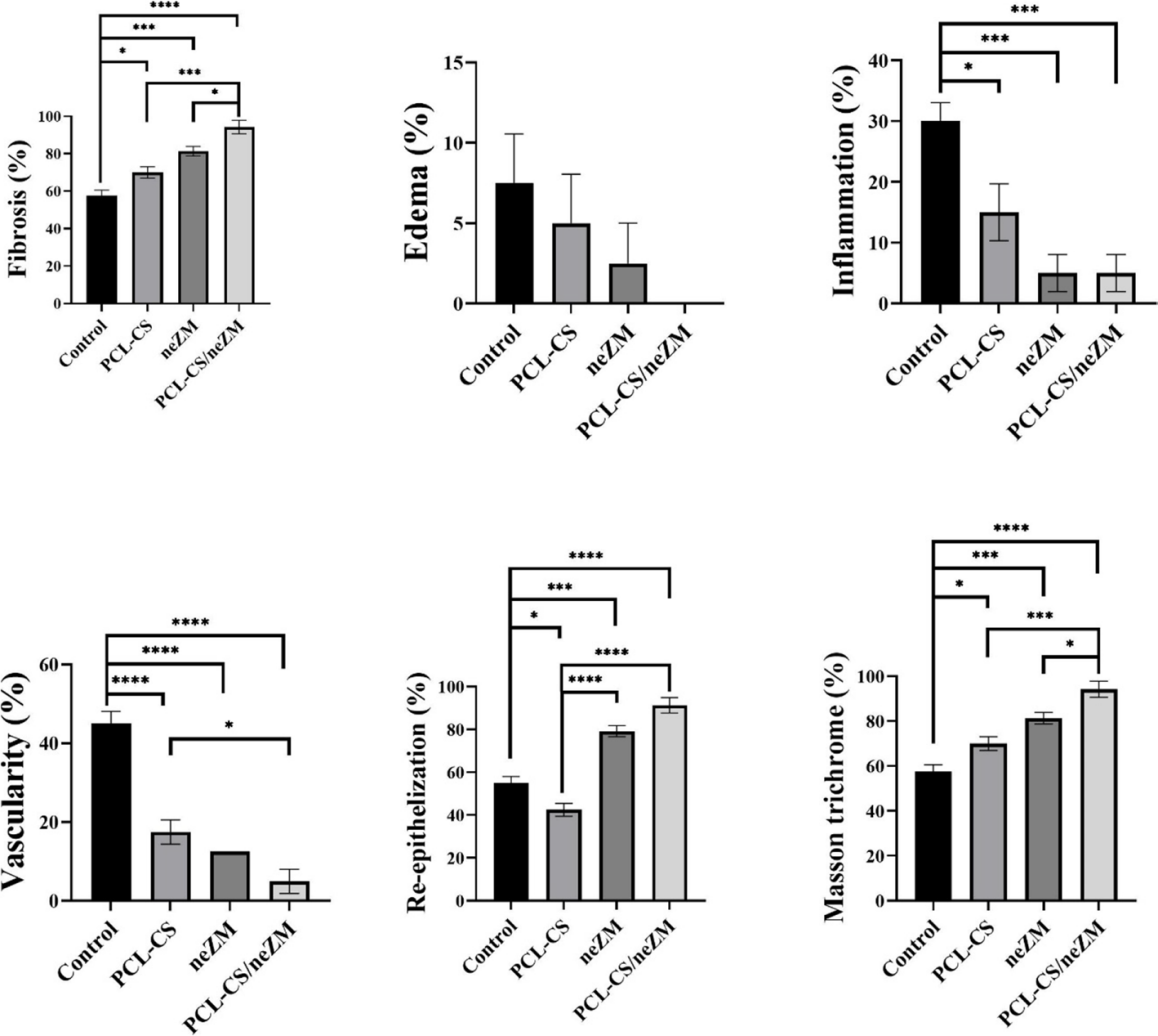
Statistical analysis of histopathological parameters of the experimental groups. The PCL-CS/neZM group represents the highest value in fibrosis parameters, re-epithelialization, and collagen deposition rate, as well as the lowest value in inflammatory parameters. *: *p*<0.05, **: *p*<0.01, and ***: *p*<0.001 shows the difference of significance level between the groups. Control: Control group, PCL-CS: PCL-CS nanofiber group, neZM: HPMC gels containing neZM group, PCL-CS/neZM: PCL-CS nanofibers coated with HPMC gels containing neZM group

Figure 13 compares the parameters of fibrosis, edema, inflammation, vascularization, re-epithelialization, and Masson trichrome blue color (indicating the amount of collagen deposition) as a percentage in the groups of this study on day 21. The statistical results revealed that the percentage of fibrosis parameters, re-epithelialization, and collagen deposition rate increased with the improvement of wound healing in the experimental groups. Hence, the PCL-CS/neZM group showed significantly higher fibrosis and collagen deposition than all of the other groups. Also, this group significantly indicates the percentage of re-epithelialization compared to the control and PCL-CS groups. Contrary to these parameters, the statistical results demonstrate that the decrease in the percentage of edema, inflammation, and vascularization are associated with improving wound healing in the experimental groups. Therefore, in the inflammation parameter, the PCL-CS/neZM, neZM, and PCL-CS groups showed a significant decrease compared to the control group, respectively. However, the reduction in the inflammatory parameters in PCL-CS/neZM group compared to the control and PCL-CS groups was significantly higher.

## Discussion

For centuries, the thyme plant has been known as an anti-inflammatory and antimicrobial plant, and there are several studies on reducing inflammatory factors and bacterial count [81, 82]. These properties introduce this plant as a suitable candidate for wound healing; despite this, there are few studies on the therapeutic effects. In this work, we prepared a hybrid novel bandage for improving wound healing by combining sustained releasing of Zataria Multiflora volatile oil (ZMVO) drug delivery and PCL-CS as an ECM biomimicry part.

In order to effectively manage wound dryness and facilitate the exchange of nutrients and waste, it is crucial to achieve optimal water absorption in nanofiber wound dressings. It's important to note that the ability of a scaffold to absorb water is influenced by various factors, including the degree of polymer crystallinity, porosity, and hydrophilicity. The water-absorbing capacity is enhanced when there is an increased amorphous region and higher porosity, which enables water to penetrate at both micro and macro levels. Research has shown that PCL/CS nanofibers exhibit greater water absorption compared to pure PCL. This can be attributed to the hydrophilic nature and lack of crystallinity in the CS chains, which makes them more inclined to interact with water molecules. Additionally, the porous structure of electrospun nanofibers containing CS facilitates water absorption through a capillary effect [83–85].

Research has provided evidence that materials with a high capacity for swelling are beneficial for promoting cell growth and attachment, as well as facilitating the movement of cells into three-dimensional scaffolds. This enhanced swelling capability allows these matrices to effectively absorb wound exudates, which are fluids that ooze from wounds, and this, in turn, helps to keep the wound area dry and safeguard it from potential infections. To explain further, when a wound dressing or scaffold can absorb wound exudates and swell appropriately, it creates an environment that is conducive to cell proliferation and attachment. This is because the cells find a favorable, moist but not overly wet environment that encourages their growth and attachment to the scaffold. Additionally, this moisture regulation helps to prevent the wound from becoming overly moist, which can be a breeding ground for infections. So, in summary, maintaining the right level of swelling in the matrix not only supports cell activity but also contributes to wound healing and protection against infections [84]. In numerous previous research investigations, it has been consistently observed that PCL/chitosan scaffolds exhibit a considerably greater ability to absorb water when compared to scaffolds made from pure PCL. Additionally, as the proportion of chitosan within the composite increases, the extent of swelling in these scaffolds has shown a noticeable enhancement [85–87].

Electrospun nanofibers need to have suitable mechanical characteristics, particularly in terms of their elongation at the point of breaking, in order to be effective for wound dressing purposes. This means that a wound dressing must satisfy specific requirements to securely cover the wound without tearing or breaking while the wound heals. According to research conducted by Mosallanezhad and colleagues, the tensile strength of nanofibers made from PCL and a blend of PCL and chitosan was found to be well-suited for wound dressing applications. In their study, PCL and chitosan were used at concentrations of 15 wt% and 3 wt%, respectively. Pure PCL nanofibers exhibited a tensile strength of approximately 1.9 ± 0.2 MPa and an elongation at break of around 54 ± 3.2%. When PCL was blended with chitosan, it led to a slight increase in tensile strength (approximately 2.2 ± 0.3 MPa) compared to pure PCL, but it resulted in a decrease in elongation at break (approximately 43 ± 3.6%) [85].

Various studies have examined the mechanical properties of PCL nanofibers and their combinations with chitosan for different applications, primarily focusing on wound dressing. In one study, pure PCL nanofibers at a 10 wt% concentration exhibited satisfactory tensile strength (7 MPa) and elongation (35%). When chitosan was added (3 wt%), the strength properties remained relatively unchanged, making them suitable for wound dressings [84]. Another study combined PCL and chitosan solutions with acetic acid at a 3:1 volume ratio. This significantly reduced UTS and elongation at break due to introducing chitosan into the PCL solution [86]. Saatcioglu et al. assessed the impact of chitosan on ligament scaffolds produced through electrospinning. Adding 1 wt% chitosan to a 10 wt% PCL solution significantly increased tensile strength, but higher concentrations of chitosan led to decreased tensile strength. Higher chitosan content improved the elongation at the point of break [87]. Surucu and Sasmazel created coaxial electrospun scaffolds with PCL and chitosan. The combination displayed higher tensile strength than PCL and chitosan fibers produced separately [88].

By evaluating the tensile strength test results of PCL/chitosan fibrous scaffolds from previous studies, it seems that the tensile strength of PCL/chitosan fibers prepared in this study is in a suitable range for wound dressing applications.

The efficiency of ZMVO in wound healing by reducing inflammation and bacterial load has been confirmed by previous researchers. For instance, Farahpour et al. synthesized an ointment containing 2 and 4% of ZMVO. They showed that ZMEO exhibits antibacterial and anti-inflammatory properties and significantly reduces wound size. The ZMEO also increased angiogenesis, collagen deposition, and re-epithelialization compared to the control group [54]. Also, Ardekani et al. fabricated different percentages of ZMVO in CS/PVA/Gelatin polymers in the form of electrospun nanofibers. Nanofiber containing 10% ZMEO showed that it can completely stop the growth of *Staphylococcus aureus, Pseudomonas aeruginosa, and Candida albicans* bacteria and is non-toxic to L929 mouse fibroblast cell line [51]. Moreover, Farahani et al. synthesized cellulose acetate/gelatin nanofibers containing ZM nanoemulsion and showed that this wound dressing can significantly improve wound healing after 22 days compared to other groups [55].

Additionally, hydroxypropyl methylcellulose (HPMC) has many applications in cosmetic materials and preparing skin creams [89, 90]. On the other hand, the efficiency of Zataria multiflora creams in clinical wound healing has been approved previously. For example, an evaluation of Zataria multiflora cream's therapeutic effect on the wound healing process of partial-thickness skin graft donor sites was conducted in a prospective, randomized, placebo-controlled trial. Enrolled patients applied twice-daily Z. multiflora cream and placebo (petrolatum ointment) on the skin graft donor site in two parts after the intervention day. There was a significant reduction in wound surface area and total score in the Z. multiflora group. In the second and third weeks, 30% and 90% of patients had fully epithelized wounds, respectively, in the Z. multiflora group. The control group's values were 3.3% and 36.7%, respectively, so the healing time was ∼9-fold in the second week and 2.45-fold in the third week in the Z. multiflora group compared with the control group. A significant increase in healing and reepithelialization was observed in the first, second, third, and fourth weeks after intervention in the Z. multiflora treatment group. This result was related to the modulation of the inflammatory phase and improving the proliferative phase [91].

Furthermore, in another study by Dashipour et al., Zataria multiflora Boiss (Avishan-e Shirazi) essential oils were incorporated into carboxymethyl cellulose films (1, 2, and 3% v/v) to evaluate their antibacterial, antioxidant, and antimicrobial properties. The results revealed that films containing the highest ZEO concentration had the highest total Phenolic (TP) content and antioxidant activity. ZEO was effective against Gram-positive and Gram-negative bacteria at all concentrations compared to control films (without ZEO) [92]. Also, Nasseri et al. reported that Zataria multiflora essential oil (ZEO) is effective at controlling fungi through solid lipid nanoparticles (SLNs) as a carrier [93]. In addition, Mahboubi and Ghazian Bidgoli showed that infections caused by Methicillin-resistant S. aureus (MRSA) were significantly reduced by Z. multiflora oil [94].

Hence, it seems that the ZMVO nanoemulsion-loaded HPMC could be considered as a suitable choice for preparing advanced drug-loaded nanogel-based creams. Therefore, it proposed that our approach to wound healing has the potential for future clinical wound healing by considering nanogel as an advanced cream and PCL-CS nanofiber as an advanced bandage.

## Conclusion

This study presents compelling evidence of the potential of a novel wound healing approach employing a combination of HPMC gel containing neZM and PCL-CS nanofibrous scaffold. The results obtained through a series of well-structured experiments and analyses offer valuable insights into the remarkable therapeutic efficacy of the PCL-CS/neZM treatment in wound healing.

The animal study, conducted on full-thickness skin wounds in rats, underscored the effectiveness of the PCL-CS/neZM treatment. Over the 7th, 14th, and 21st days, wounds treated with PCL-CS/neZM consistently demonstrated accelerated wound closure, outperforming both the individual treatment groups and the control group, which used common sterile gas bandaging. Histological assessments, particularly on the 21st day, revealed substantial improvements in key wound healing parameters, including reduced edema, inflammation, and vascularity, coupled with increased fibrosis, re-epithelialization, and collagen deposition. This not only signifies enhanced wound closure but also points to improved tissue regeneration and scar quality.

The results of this study are promising and highlight the potential of the PCL-CS/neZM treatment as an effective modality for enhancing wound healing processes.

## Data Availability

All data generated during the current study are available from the corresponding author upon reasonable request.
